# The relationship between attitudes towards pregnancy and contraceptive continuation: Results from a longitudinal study of married women in India

**DOI:** 10.1371/journal.pone.0229333

**Published:** 2020-02-25

**Authors:** Elizabeth Tobey, Aparna Jain, Arupendra Mozumdar

**Affiliations:** 1 Population Council, Washington, DC, United States of America; 2 Population Council, New Delhi, India; Johns Hopkins University Bloomberg School of Public Health, UNITED STATES

## Abstract

To understand the relationship between pregnancy intentions and contraceptive use, a growing body of research has begun to examine various domains of women's attitudes towards pregnancy, acknowledging that these attitudes may contradict one another, and women may be ambivalent. This study examines pregnancy ambivalence and assesses the relationship between attitudes towards pregnancy and contraceptive continuation after nine months among a sample of women in Odisha and Haryana, India. Data come from a longitudinal study of married women age 15–49 who began using a modern reversible method of contraception at the time of study enrollment. To assess their cognitive attitudes (beliefs/knowledge) towards pregnancy, women were asked “how important is it you to avoid a pregnancy now?” To assess their affective attitudes (feelings/emotions), women were asked about their agreement with the statement: “If I found out I was pregnant in the next several weeks, I would be happy.” A joint, 4-category measure combining these cognitive and affective attitudes towards pregnancy was created to measure concordance and ambivalence in attitudes towards pregnancy. Multivariate random-effects logistic regression models were employed to examine the relationship of these two measures with method-specific contraceptive continuation nine months later. Two models were conducted, one with the two attitude variables included independently and the second with the joint, 4-category measure included. Results showed that affective and cognitive attitudes were both significantly associated with continuation, but that there were no significant differences between those that were ambivalent and those whose attitudes were concordantly anti-pregnancy. This study suggests that attitudes towards pregnancy are multifaceted and both cognitive and affective attitudes towards pregnancy may play an important role in contraceptive use in India.

## Introduction

In India, the most commonly used method of contraception is female sterilization, with 36 percent of married women age 15–49 using the method, constituting 75 percent of modern method use [1]. While this is due in part to a long history of inclusion of female sterilization as part of India’s family planning program [2], it may also be due in part to a concern about side effects of hormonal methods of contraception [3]. Many women who ultimately use female sterilization for limiting births do not use other modern methods of contraception prior (Sebastian 2010). One study from Odisha state demonstrated that among women who had undergone sterilization, 72 percent did not use contraception between their last childbirth and their sterilization, despite this interval being a mean duration of 17 months [4]. Given this limited use of modern reversible methods in a context where women can readily choose female sterilization to limit childbearing, there is a need to better understand the attitudes and behaviors of women who use modern reversible methods of contraception in India, including their attitudes around future pregnancy. This paper aims to examine women’s attitudes towards pregnancy and the association of these attitudes with contraceptive continuation among a sample of modern reversible method users in Odisha and Haryana, India.

Global rates of unintended pregnancy have decreased but remain high, and nearly half of pregnancies are unintended [5]. Unintended pregnancy is widely used as an indicator of women’s and couples’ ability to meet their reproductive goals [6–7], and is also linked to negative health outcomes for mothers and children [8]. A significant body of research has been conducted to determine the risk factors of unintended pregnancies, as well as the risk factors of having an unmet need for family planning (wanting to delay or limit childbearing but not using a contraceptive method), a risk factor for unintended pregnancy [9]. However, the constructs of unintended pregnancy and unmet need rely on the assumptions that pregnancy intentions are concrete and binary, and that women who have an unmet need or are at risk of an unintended pregnancy will use contraception if given the opportunity. This is often not the case, which has sparked many efforts to examine how to better measure fertility preferences [10–13] and understand their relationship with contraceptive use [14–19], with the goal of being able to better meet the family planning needs of women.

Unintended pregnancy, measured as the percentage of births in the last five years that were unwanted or mistimed, is measured retrospectively, and thus subject to recall bias and post-rationalization, in which respondents adapt to their recent child, or are reluctant to describe recent births as being mistimed or unwanted [20–23]. Unmet need, measured as the percent of women currently married or in union and who desire to delay or limit childbearing but are not using a contraceptive method, is a construct that is not directly asked of women [24]. These measures do not allow room for mixed feelings, or ambivalence towards pregnancy, which research has long shown to be quite common in the United States [25–26]. Several studies have demonstrated ambivalence is common in global contexts as well, including India [27], Indonesia [28], Honduras [29], Morocco [21], Burkina Faso, Ghana, and Kenya [30]. With the goal of understanding how these preferences relate to contraceptive behaviors, a growing body of research has begun to examine multiple domains of women’s attitudes toward pregnancy, acknowledging that these attitudes may contradict each other, change over time, and women may be ambivalent (that is, have mixed or contradictory feelings) [25, 31–32].

Studies have generally found that ambivalent women are less likely to use contraceptives than women whose express concordant pregnancy-avoidant attitudes. In the United States, Moreau et al. [17] combined measures of desire to become pregnant and desire to avoid pregnancy and found that those who were ambivalent were more likely to report inconsistent contraceptive use than women who had concordant anti-pregnancy attitudes. Higgins et al. [18] found that women who were ambivalent towards pregnancy were less likely to use contraception than those who were concordantly anti-pregnancy. Frost et al. [19] similarly found that ambivalence was associated with both nonuse of contraception and gaps in contraceptive use. Outside of the United States, Huber et al. [14] asked a cohort of rural Malawian women two questions about pregnancy desires: how strongly a woman wanted to become pregnant in the next year and how strongly a woman wanted to avoid a pregnancy in the next year. Results showed that those who had a reported desire to become pregnant but also wanted to avoid a pregnancy (which they categorized as ambivalent) did not differ from those who were concordantly pro-pregnancy in terms of contraceptive use, while those who did not desire to become pregnant but also did not want to avoid pregnancy (indifferent) were more likely to use contraception. They conclude that the lack of a desire to become pregnant was a more important contributor to contraceptive use than a desire to avoid pregnancy.

Ambivalence has most often been measured as a midpoint between two ends of a pregnancy desire spectrum [33], but two recent studies have examined the interactive effect of two different domains of pregnancy attitudes on contraceptive use: cognitive and affective attitudes [15–16]. Cognitive attitudes capture a person’s belief or knowledge, while affective attitudes capture emotions and feelings. A woman can believe it is important to avoid pregnancy (cognitive attitude), but also feel happy if she were to get pregnant (affective attitude), or vice versa. This woman would be considered ambivalent toward pregnancy. Jones [15] and Yoo et al. [16] found that cognitive attitudes about the importance of avoiding pregnancy played a more important role in consistent contraceptive use over time than affective attitudes among women in the United States.

In this paper, we aim to examine cognitive and affective attitudes towards pregnancy, including ambivalence, among a sample of women in Odisha and Haryana states in India. We assess the associations between cognitive and affective attitudes and contraceptive method continuation both independently and as a joint measure to examine differences between those who are ambivalent and those with concordant attitudes. Given the context of high rates of sterilization and limited use of modern reversible methods of contraception in India, examining the relationship between attitudes towards pregnancy, including ambivalence, and contraceptive continuation could be especially useful in this setting.

### Indian context

In India, the rate of unintended pregnancy is 70.1 per 1000 women age 15–49—approximately half (48%) of all pregnancies [34]—despite 54% of married women age 15–49 using any method of contraception and 48% using a modern method (44% of all women age 15–49 use any method of contraception, while 38% of all women use a modern method) [1]. In Odisha and Haryana, the two states in which this study was implemented, the contraceptive prevalence is slightly higher than that of India overall, as 57% of married women in Odisha and 64% of those in Haryana use any method of contraception. However, in Odisha, the modern contraceptive prevalence is 45% among married women age 15–49, while in Haryana it is 59% [1]. In Odisha, 14% of married women have an unmet need for family planning, while 9% of women in Haryana have an unmet need, compared to a national average of 13 percent. In Odisha, the median age at marriage is 19.9 years, and in Haryana, it is 19.5 years among women age 20–49. In each state, the total fertility rate is 2.1 children per woman, replacement level fertility, and just under the national average of 2.2 children per woman [1].

The government of India has committed to increasing access to and demand for modern reversible methods of family planning [35], as 75% of married women who use modern contraceptives use female sterilization [1]. Use of female sterilization, while still high in Odisha and Haryana, is slightly less than the national average: female sterilization makes up 62% of modern method use among married women in Odisha and 64% of modern method use among married women in Haryana [1]. As demand for and use of reversible methods increases throughout the country, an understanding of the dynamics of reversible contraceptive use, including how attitudes about pregnancy affect contraceptive behavior, is needed.

## Methods

### Data

The analysis presented in this study is secondary data analysis of a larger 12-month longitudinal study on contraceptive use dynamics of married women aged 15–49 in India. Respondents who began a new episode of intrauterine device (IUD, including interval and postpartum [PPIUD]), injectable, or oral contraceptive pill (OCP) use were enrolled into the study within 30 days of beginning the method. A new episode of use includes new users of family planning or those who had used a family planning method in the past but were not using one right before the method selected at enrollment. Enrollment occurred in Haryana and Odisha states from December 2016 through October 2017. Respondents were interviewed at enrollment into the study and at 3-, 6-, and 12-month follow-up, regardless of their contraceptive use status at the time of each follow up. Questionnaires were translated into Hindi and Odia and then back translated into English so discrepancies could be reconciled. In Haryana, all respondents were enrolled through accredited social health activists (ASHAs) at the community level. In Odisha, PPIUD users were enrolled by the research team at government health facilities, interval IUD and OCP users were enrolled primarily through ASHAs at the community level, and injectable users were enrolled by the research team primarily at non-governmental organization (NGO) facilities. Though ASHAs assisted in enrolling women into the study, they did not conduct interviews–female investigators that were part of the research team conducted interviews at each of the four time points. Sample sizes were calculated to enable estimation of discontinuation rates for IUD, injectable, and OCP users, and 2,699 women were enrolled into the study. At the 12-month follow-up interview, 2,441 respondents were interviewed, for a 90.4% follow-up rate.

The questionnaires asked respondents about a range of information, including demographic characteristics, fertility intentions, contraceptive use, quality of care at the time of adoption of most recent method, experience of side effects, reasons for discontinuing contraceptive methods, and contraceptive switching behaviors.

Ethical approval was obtained from the Population Council Institutional Review Board, the ethics committee of the Government of Odisha, and by district authorities in selected districts in Haryana. Written consent was obtained from all respondents before each survey.

### Sample population

As this paper describes a secondary data analysis from a larger study of contraceptive use dynamics, the sample population for this analysis was determined by data availability for the key measures in this study. Pregnancy attitudes were asked in the 3-month survey among women who reported wanting to have children in the future (n = 706). Women who wanted to have children within the next year were excluded from the sample as they would be more likely to discontinue their method due to the desire to become pregnant (n = 660). The sample was limited to modern reversible contraceptive users (n = 577) who answered pregnancy attitude questions at 3-month and those not lost to follow-up at 12-month (n = 562).

### Measures

#### Dependent variable

The dependent variable is method-specific continuation of a modern contraceptive method (IUD, injectable, OCPs, or condoms) after nine months. This time frame was chosen because the women included in this sample wanted to have children in the future but did not want to have children within the next year, and thus contraceptive use after 9 months is an important marker of these women’s ability to meet their fertility preferences. Method-specific contraceptive continuation includes those who continued their original modern contraceptive method (coded as 1). Those who had switched to a different modern method, a traditional method (including abstinence, withdrawal, and rhythm), or were not using any method of family planning after nine months were considered discontinuers of their contraceptive method and were coded as 0.

#### Independent variables

The main independent variables are measures of affective and cognitive attitudes toward pregnancy. Affective attitudes were captured in the survey by agreement with the statement: “If I found out I was pregnant in the next several weeks, I would be happy.” Response options were on a 4-category Likert scale: strongly agree, agree, disagree, strongly disagree. This scale was assessed as an ordinal, 4-category variable and was dichotomized for inclusion in multivariate models: those who agreed and strongly agreed were coded as “would be happy if pregnant,” and those who disagreed and strongly disagreed were coded as “would be unhappy if pregnant.” Cognitive attitudes were measured responses on a 10-point scale to: “How important is it to you to avoid a pregnancy now?”, with 10 being extremely important. This scale was assessed as an ordinal, 10-category variable and was also dichotomized into two groups for inclusion in multivariate models: those who answered 6–10 (important to avoid pregnancy) and those who answered 1–5 (not important to avoid pregnancy.)

Adapting the approach and terms from prior research [14–16], a 4-category measure of attitudes toward pregnancy was constructed by combining the measures of cognitive and affective attitudes: pro-pregnancy (would be happy if she found out she was pregnant/not important to avoid pregnancy), anti-pregnancy (would be unhappy if she found out she was pregnant/ important to avoid pregnancy), positive ambivalent (would be happy if she found out she was pregnant/ important to avoid pregnancy) and negative ambivalent (would be unhappy if she found out she was pregnant/not important to avoid pregnancy) (Table 1).

**Table 1:** Attitudes towards pregnancy.

		Affective attitude (feelings/emotions)	
		Would be unhappy if pregnant	Would be happy if pregnant
**Cognitive attitude (belief/knowledge)**	**Important to avoid pregnancy**	Anti-pregnancy	Positive ambivalence
	**Not important to avoid pregnancy**	Negative ambivalence	Pro-pregnancy

Covariates of interest that were considered include: age, education, number of living children, religion, employment, desired timing of next birth, and type of contraceptive method used at the time of the interview.

### Analysis

Descriptive statistics were calculated for respondent characteristics, dependent and independent variables. Simple logistic regression models were conducted to examine associations between respondent characteristics and method-specific contraceptive continuation. A random effects multivariate logistic regression model (Model 1) was employed to assess the relationship between cognitive and affective attitudes towards pregnancy (each included in the model independently) and method-specific continuation. Covariates included in the model based on theoretical importance and bivariate significance were age, number of living children, education, previous use of a modern method, and method used. A second model (Model 2) was conducted with the 4-category joint measure of attitudes towards pregnancy to assess differences between those who were ambivalent and those who had concordant attitudes towards pregnancy. The likelihood ratio test was employed to assess whether inclusion of the joint measure improved model fit. All analyses were conducted using Stata version 15.

## Results

Table 2 presents background characteristics of respondents at enrollment (n = 562). Two-thirds of respondents were under the age of 25 (67%), one-quarter were between the ages of 25–29 (26%) and eight percent were age 30 or older. Most had attended secondary school or higher (63%), while 25% had attended primary or middle school and 13% never attended. In this sample, most respondents were from Odisha state (79%). Most had had one living child (80%), though two percent did not have children and 18% had two or more. The majority were Hindu (94%), while 6% were Muslim or another religion. Most worked as housewives (94%). Thirteen percent wanted a child in one-two years, 64% in two or more years, and 22% were unsure. Nearly half had used a modern contraceptive method prior to enrollment (43%). At the three-month survey, 40% of respondents were using the IUD, 8% were using the injectable, 50% were using OCPs, and 3% were using condoms.

**Table 2:** Respondent characteristics (n = 562).

	Percent	N
**Age**		
17–19 years	7.3	41
20–24 years	59.3	333
25–29 years	25.6	144
30+ years	7.8	44
**Education**		
Never attended school	12.8	72
Primary/middle	24.7	139
Secondary/higher	62.5	351
**State**		
Odisha	79.4	446
Haryana	20.6	116
**Number of living children**		
Zero	2.0	11
One	79.7	448
Two or more	18.3	103
**Religion**		
Hindu	93.8	527
Muslim/other	6.2	35
**Employment**		
Housewife	94.1	529
Other	5.9	33
**Fertility preferences**		
Wanted child within 1–2 years	13.3	75
Wanted child in more than two years	64.4	362
Undecided about timing	22.2	125
**Previous use of modern method**		
No	56.9	320
Yes	43.1	242
**Modern method type**		
IUD	39.7	223
Injectable	7.5	42
OCPs	49.5	278
Condom	3.4	19
**Attitudes towards pregnancy**		
Anti-pregnancy	71.5	402
Pro-pregnancy	5.7	32
Negative ambivalent	5.2	29
Positive ambivalent	17.6	99
**Continued using method after nine months**		
Yes	77.2	434
No	22.8	128
Discontinued contraception	12.8	72
Switched to modern method	7.1	40
Switched to traditional method	2.8	16

Applying the four attitudinal categories from previous literature, 72% of respondents would be categorized as anti-pregnancy, 6% as pro-pregnancy, 5% as negative ambivalent, and 18% would be categorized as positive ambivalent. In total, 23% were ambivalent (including both positive and negative ambivalence).

After nine months, 77% of respondents were using the same modern method of contraception. The 23% that discontinued was comprised of 13% that discontinued contraception altogether, 7% that switched to another modern method, and 3% that switched to a traditional method.

**Fig 1:**
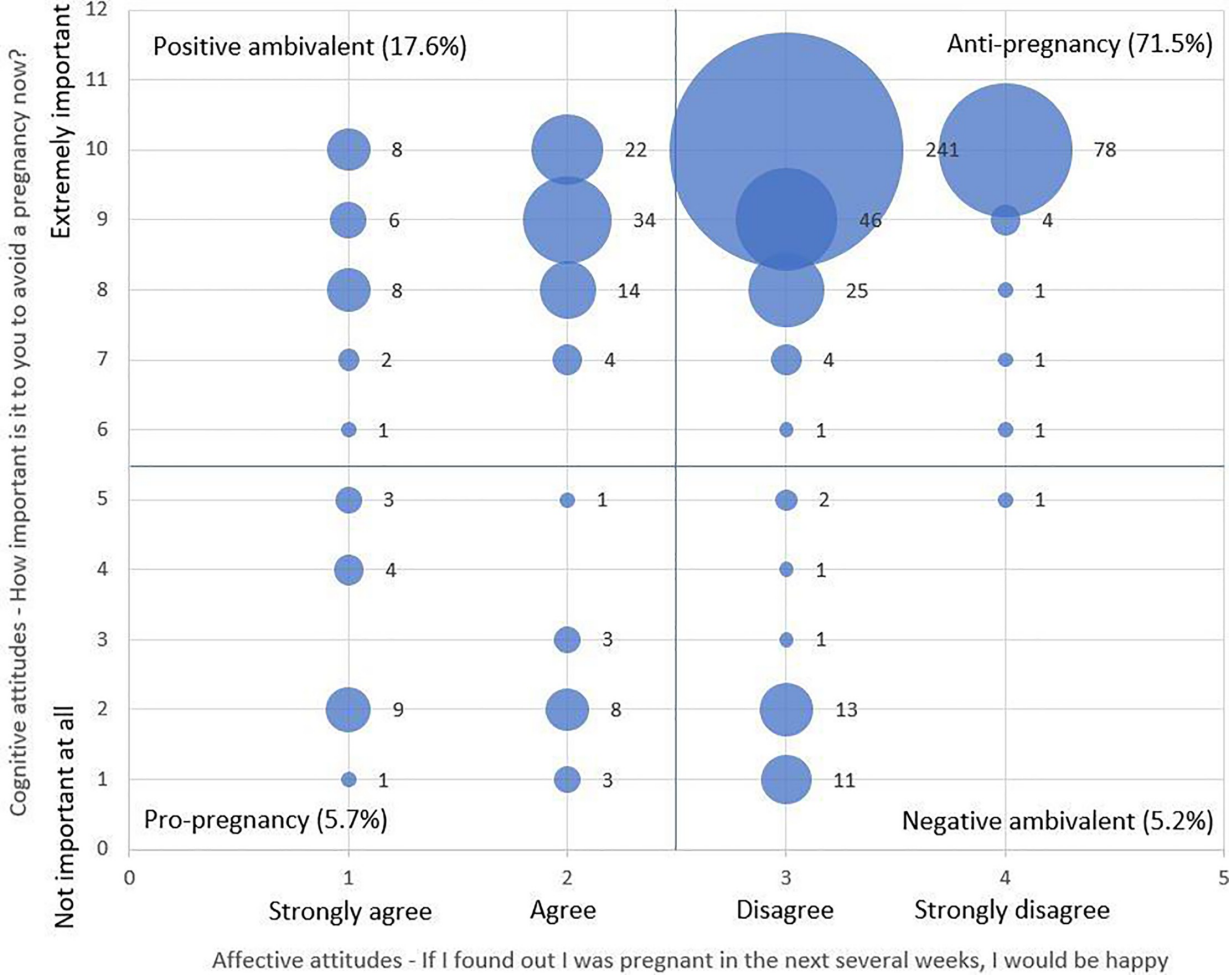
Distribution of women’s responses to cognitive and affective attitude questions (n = 562).

Fig 1 presents the distribution of women’s responses to both the cognitive and affective attitude questions. Most women (77%) reported that they disagreed or strongly disagreed with the statement “If I found out I was pregnant in the next several weeks, I would be happy,” and most (87%) reported that it was extremely important for them to avoid a pregnancy now (reported at least 8 on the scale of 1–10).

Results of the multivariate random effects model of attitudes on contraceptive continuation (adjusted for age, parity, education, previous use of modern contraception and type of contraceptive method) showed that attitudes were significantly associated with contraceptive method continuation after 9 months (Table 3).

**Table 3:** Unadjusted and adjusted odds ratios and confidence intervals of method-specific contraceptive continuation after 9 months (n = 562).

			Model 1: Independent cognitive and affective attitude measures		Model 2: Joint cognitive and affective attitude measure	
	Unadjusted odds ratio (OR)	95% CI	Adjusted odds ratio (AOR)	95% CI	Adjusted odds ratio (AOR)	95% CI
**Age**						
17–24 years	Ref		Ref		Ref	
25–40 years	1.19	(0.78–1.83)	1.37	(0.85–2.19)	1.32	(0.82–2.12)
**Number of living children**						
Zero or one child	Ref		Ref		Ref	
Two or more children	0.79	(0.49–1.30)	0.72	(0.41–1.26)	0.74	(0.42–1.31)
**Education**						
No schooling	Ref		Ref		Ref	
Primary/middle	1.14	(0.56–2.33)	1.08	(0.52–2.25)	1.05	(0.50–2.18)
Secondary or higher	0.80	(0.43–1.48)	0.73	(0.38–1.42)	0.73	(0.38–1.42)
**Previous modern method use**						
No	Ref		Ref		Ref	
Yes	0.93	(0.62–1.38)	1.05	(0.68–1.61)	1.02	(0.66–1.57)
**Method used at time of interview**						
Long-acting reversible (IUD)	Ref		Ref		Ref	
Short-acting reversible (injectables, oral contraceptive pill, condoms)	1.01	(0.67–1.51)	0.99	(0.64–1.51)	1.01	(0.66–1.55)
**Cognitive attitudes**						
Unimportant to avoid pregnancy	Ref		Ref			
Important to avoid pregnancy	2.48*	(1.41–4.32)	2.00*	(1.11–3.61)		
**Affective attitudes**						
Would be happy if pregnant	Ref		Ref			
Would be unhappy if pregnant	2.09*	(1.35–3.22)	1.82*	(1.14–2.90)		
**Joint measure**						
Pro-pregnancy	Ref				Ref	
Anti-pregnancy	5.34*	(2.54–11.2)			4.96*	(2.35–10.5)
Negative ambivalent	4.93*	(1.58–15.4)			4.43*	(1.40–14.1)
Positive ambivalent	3.61*	(1.57–8.27)			3.32*	(1.43–7.68)

*p<0.05

Note: anti-pregnancy: would be unhappy if pregnant, important to avoid pregnancy

Pro-pregnancy: would be happy if pregnant, not important to avoid pregnancy

Negative ambivalent: would be unhappy if pregnant, not important to avoid pregnancy

Positive ambivalent: would be happy if pregnant, important to avoid pregnancy

Model 1 presents the results of the model with cognitive and affective attitudes included independently. In this model, cognitive and affective attitudes were both significantly associated with method-specific continuation after 9 months. Those who reported that it was important to avoid pregnancy (cognitive attitude) were 2.00 (95% confidence interval [CI]: 1.11–3.61) times more likely to have continued using the same modern method of contraception compared to those who reported that it was unimportant to avoid pregnancy. Women who reported that they would be unhappy if they found out they were pregnant were 1.82 (95% CI: 1.14–2.90) times more likely to continue using their method after 9 months compared to those who agreed that they would be happy.

Model 2 presents the results of the model with the joint measure of cognitive and affective attitudes towards pregnancy. Women who had anti-pregnancy attitudes (would be unhappy if they found out they were pregnant/important to avoid pregnancy) were 4.96 times more likely (95% CI: 2.35–10.5) to continue to use their contraceptive method 9 months later than those who were pro-pregnancy (would be happy if she found out she was pregnant/ not important to avoid pregnancy). Women who had negative ambivalent attitudes (would be unhappy if she found out she was pregnant/not important to avoid pregnancy) were significantly more likely to continue their contraceptive method 9 months later than those who were pro-pregnancy (AOR = 4.43, 95% CI: 1.40–14.1). Women who were positively ambivalent (would be happy if she found out she was pregnant/ important to avoid pregnancy) were more likely than likely than pro-pregnancy women to continue using their method 9 months later (AOR: 3.32, 95% CI: 1.43–7.68). In post-estimation linear combination tests, there were no significant differences between those who were negative ambivalent and positive ambivalent (AOR = 1.33, 95% CI: 0.48–3.71), between those who were anti-pregnancy and positive ambivalent (AOR = 1.49, 95% CI: 0.48–3.71), or between those who were anti-pregnancy and negative ambivalent (AOR = 1.12, 95% CI = 0.44–2.88).

There were no significant differences in Model 1 or Model 2 in terms of method continuation 9 months later by age, parity, education, previous use of a modern method, or method type used after adjusting for attitudes toward pregnancy. The likelihood ratio test was used to assess the differences between the two models. The test had a *p*-value of 0.084 and was not significant, indicating that using the joint measure in Model 2 did not significantly improve the fit of the model to the data.

### Limitations

This study had several limitations. First, because this is secondary data analysis from a larger study on contraceptive use dynamics, this analysis was only able to examine married women who were using a contraceptive method at the time the questions about attitudes towards pregnancy were asked. Thus, there may be limited variation in attitudes towards pregnancy given that everyone had begun using a method within a month of the start of the study and thus wanted to prevent pregnancy at that time. Additionally, women who did not report wanting to have children in the future were not asked about their affective attitudes towards pregnancy and were excluded from analysis. These results are therefore not generalizable to unmarried women, those not using a contraceptive method, or women who want to limit childbearing. Additionally, perhaps due in part to the limited variation in attitudes towards pregnancy and fertility preferences, desired timing of next birth was highly correlated with attitudes towards pregnancy in this population, so it was not included in the model. However, by removing from the analysis those who wanted to have a child within the next year, the sample was limited to women who wanted to prevent pregnancy during the observation period.

Second, enrollment of respondents differed by state and by method used at enrollment. In Odisha, respondents were enrolled at different types of facilities or at the community level based on the method they chose, and all respondents in Haryana were recruited through ASHAs at the community level. Women interviewed at facilities may have experienced more social desirability bias in their responses to the questions than those who were interviewed at their home. Given the variation in enrollment strategies, results are not necessarily representative of all users of modern reversible methods in Odisha and Haryana, India.

Lastly, method-specific contraceptive continuation was used as the outcome in this study, excluding women who switched to other modern methods or traditional methods of contraception from the numerator. While switching methods should be considered a positive outcome for women who want to continue preventing pregnancy but find their current method unsuitable [36], in this study there was not enough variation in the outcome variable of modern contraceptive continuation, so method-specific continuation was used instead.

## Discussion

This study examines pregnancy ambivalence and the relationship between cognitive and affective attitudes and method-specific contraceptive continuation after 9 months among married women in India. Results demonstrate that approximately one quarter of contraceptive users who would like to have children in the future were either positively or negatively ambivalent about pregnancy (23%), and that both cognitive (beliefs and knowledge) and affective (feelings and emotions) attitudes toward pregnancy were significantly associated with method-specific contraceptive continuation 9 months later. These results support a growing body of research, largely from the United States, demonstrating that women’s attitudes towards pregnancy are multifaceted, that cognitive and affective attitudes can contradict each other, and that more nuance is warranted in the measurement of pregnancy and fertility desires and intentions [14–16].

Among this sample of women from Odisha and Haryana, affective and cognitive attitudes towards pregnancy are independently important predictors of method-specific contraceptive continuation. The results of Model 2, which combined cognitive and affective attitudes into a joint measure, showed that women who were anti-pregnancy (would be unhappy if pregnant, important to avoid pregnancy), positive ambivalent (would be happy if pregnant, important to avoid pregnancy) and negative ambivalent (would be unhappy if pregnant, not important to avoid pregnancy) were all significantly more likely than women who were pro-pregnancy (would be happy if pregnant, not important to avoid pregnancy) to continue using their contraceptive method after 9 months. However, there were no significant differences between those who were positive ambivalent, negative ambivalent, and anti-pregnancy, suggesting that women who hold either the cognitive attitude that it is important to avoid pregnancy or the affective attitude that they would be unhappy if they were pregnant are just as likely to continue their contraceptive method as women who held both anti-pregnancy attitudes. These findings suggest that in this context, both attitudes are independently important factors in contraceptive continuation and should be included in counseling messages and explored further in future research. Both attitudes should be captured in contraceptive counseling, as women who express either an affective or cognitive attitude against becoming pregnant in the near future may be candidates for voluntary, reversible family planning for healthy birth spacing. These measures should also be included in future studies on attitudes towards pregnancy. Future research should continue to test the relationship between contraceptive continuation and both cognitive and affective attitudes towards pregnancy, including the joint measure to assess whether there are differences between those who are ambivalent and those who have concordant attitudes.

These findings, that both cognitive and affective attitudes towards pregnancy were significantly associated with contraceptive use, contrast with studies from the United States, in which cognitive attitudes were found to be more important than affective attitudes in consistent use of contraception [15–16]. There are several possible reasons for why affective attitudes were also significantly associated with continuation in this population but not in the studies from the United States. First, the current study assessed contraceptive continuation among women who were already using modern reversible contraception at the time of the first survey. These women had already made a rational, cognitive choice to use a contraceptive method, so affective feelings about happiness towards pregnancy may be more significantly associated with deciding to continue using a modern method once a decision has been made to begin using a method. Additionally, some of these women were interviewed in a healthcare setting where they had obtained their contraceptive method. This may lead to more consistent reports of the cognitive importance to avoid pregnancy, leading to relatively more variability in the affective measure than in the cognitive measure, and thus a greater ability to detect associations between affective attitudes.

Additionally, affective attitudes have been shown to be a more important factor in other health behaviors than cognitive attitudes [37], particularly when those behaviors have been experienced in the past and are associated with a strong change in affect/emotion [38]. In one U.S. study, three-quarters (76%) of women had not yet had children [16], and in the other this proportion was not stated [15]. The vast majority of respondents in the present study had at least one child and therefore had experienced affective attitudes towards pregnancy in the past. Though the number of living children was adjusted for in the final model of each study, this may be an important factor in the relative importance of affective attitudes towards pregnancy, as most respondents have experienced birth and all of the physical, familial, and emotional changes that accompany a pregnancy and birth of a child.

Lastly, this study took place in a very different context than the previous studies, in two largely rural states in India. Ambivalence towards pregnancy in this population has not been well examined, though three studies in other states in India have found that women’s attitudes towards pregnancy can be complex and changing: while many women prospectively classified births as unwanted, after giving birth, they retrospectively classified them as wanted [27, 22–23]. While this may be due to reluctance to describe an existing child as being initially unwanted, another possible explanation for this finding could be that these women’s reports of intention at one or both time points were required to fit into a binary categorization of intention and were not sufficiently capturing the possibility of ambivalence or mixed cognitive and affective attitudes towards pregnancy. Future research could explore whether cognitive or affective attitudes towards pregnancy are more likely to be consistent before and after experiencing a pregnancy to examine this phenomenon further. Given the context of high levels of female sterilization among women who want to limit childbearing in India, women who choose reversible methods may be more likely to be ambivalent, even though they still have a met need for family planning, than those who choose sterilization, and thus it is important to further explore pregnancy ambivalence and how attitudes may change over time in this context.

Many in the family planning and reproductive health field are acknowledging the limits of unintended pregnancy and unmet need in capturing and measuring a woman’s pregnancy desires and her ability to fulfill those desires. A recent commentary by Potter et al. [6] argued that unintended pregnancy is not an ideal indicator for women’s and couples’ ability to determine whether and when to have children, in large part because it is a binary classification for a concept that is far more nuanced. They argue health providers, programmers, and policymakers, rather than simply aiming to reduce the number of unintended pregnancies, should instead frame family planning interventions as enabling women to choose whether and when they have children. Findings from this study support the need for measures of pregnancy desires that are more nuanced than unintended pregnancy and unmet need, though additional research is needed on how to best incorporate questions on cognitive and affective attitudes towards pregnancy into routine counseling.

Future research on the prevalence of ambivalence and its effects on contraceptive use in India is warranted as the government aims to increase demand for and use of modern reversible contraceptive methods. Suggested future research includes examining qualitatively women’s cognitive and affective attitudes towards pregnancy, their perceived control over pregnancy planning, and how their attitudes map on to fertility intentions, as some mixed-method studies have shown that quantitative interviews may overestimate ambivalence towards pregnancy when compared to qualitative interviews [39–40]. Additionally, further research is needed on how attitudes towards pregnancy interact with other relevant considerations, including side effects, quality of care, and relationship dynamics, to affect contraceptive and birth outcomes.

These results show that attitudes toward pregnancy are multifaceted, can be contradictory, and can be more nuanced than current measures of intentions allow for. Understanding affective and cognitive attitudes toward pregnancy may help providers predict who is likely to stop using their method in the future and provide counseling messages and ask questions to determine what methods might be most appropriate for these women.

## Supporting information

S1 File Survey questions used in the study. (DOCX)
