# Supplementary material for: The relationship between attitudes towards pregnancy and contraceptive continuation: Results from a longitudinal study of married women in India
Source: PLoS One. 2020 Feb 25;15(2):e0229333. doi: 10.1371/journal.pone.0229333 (PMC7041815; doi:10.1371/journal.pone.0229333)
Supplement: S1 File — (DOCX) [file pone.0229333.s001.docx]

**Enrollment questionnaire**

|  | State code | Odisha  Haryana | 2  3 |  |
| --- | --- | --- | --- | --- |
| PE1 | How old were you on your last birthday? | Record age in completed years | \|  \|  \| \| --- \| --- \| | **STOP interview if younger than 15 or older than 49** |
| PE2 | Are you currently married? | Yes  No | 1  2 | **End Interview** |
| A1 | Have you ever attended school? | Yes  No | 1  2 | **A3** |
| A2 | What is the highest standard that you have completed?  INS: Code exact number of years of schooling | ……….Standard  BA/BSc/BCom/Graduation  Post graduate  Technical education after 10th Class  Professional education  Other (specify) ___________  ½ | \|  \|  \| \| --- \| --- \|   15  17  13  18  77 | **A5** |
| A8 | What is your religion? | Hindu  Muslim  Others (specify) _________ | 1  2  7 |  |
| A11 | What is your occupation? | Housewife  Petty business  Business including retailer shop  Farmer (own land)  Agricultural laborer  Factory/Production worker  Office attendant (peon/security staff)  Teacher/ clerk (other white collar job)  Managerial/Professional student  Other (specify) ___________________ | 01  02  03  04  05  06  07  08  09  77 | **A15**  **A14**  **A15** |

| C4 | Other than the current method, have you ever used any method to delay or avoid getting pregnant? | Yes  No | 1  2 | C6 |
| --- | --- | --- | --- | --- |
| C5 | Name all the methods that you have used in the past.  ***Probe: Any other answer?***  ***INS: Multiple answers possible. Code all that apply*** | IUD/Copper-T  Injectables  Pill  Male Condom  Female Condom  Standard Days Method  LAM  Rhythm Method  Withdrawal  Abstinence  Emergency contraceptive pill  Spermicide  Other(Specify)…………………… | A  B  C  D  E  F  G  H  I  J  K  L  X |  |

**3-month questionnaire**

| B11 | Do you have any living children? | Yes  No |  | **B18** |
| --- | --- | --- | --- | --- |
| B12 | How many living children do you have? | Number of children… | \|  \|  \| \| --- \| --- \| |  |
| B16 | a) Would you like to have more children? | Yes  No  Undecided | 1  2  3 | B20  B20 |
|  | c) How long would you like to wait from now before the birth of another child? Would you like the birth of your next child to take place within the next year, within 1 to 2 two years from now or more than two years from now? | Within the next year  Within 1 to 2 years from now  More than 2 years from now  Undecided | 1  2  3  4 |  |
| B17 | ***For the following statements, please tell me if you strongly agree, agree, disagree or strongly disagree.***  ***If I found out I was pregnant in the next several weeks….*** | | | |
|  | a) I would be happy. | Strongly agree  Agree  Disagree  Strongly disagree | 1  2  3  4 |  |
| B18 | a) Would you like to have a child? | Yes  No | 1  2 | B20 |
|  | c) How long would you like to wait from now before the birth a child? Would you like the birth of a child to take place within the next year, within 1 to 2 two years from now or more than two years from now? | Within the next year  Within 1 to 2 years from now  More than 2 years from now  Undecided | 1  2  3  4 |  |
| B19 | ***For the following statements, please tell me if you strongly agree, agree, disagree or strongly disagree.***  ***If I found out I was pregnant in the next several weeks….*** | | | |
|  | a) I would be happy. | Strongly agree  Agree  Disagree  Strongly disagree | 1  2  3  4 |  |
| C1 | What method were you using *3 months* ago? | Copper-T/IUD  Injectable  Pill | 1  2  3 |  |
| C2 | Are you currently using _____________ (*insert name of method identified in C1*)?  **INT: Check this against your records** | Yes  No | 1  2 | Skip to section D |
| C3 | Are you currently using a different family planning method? | Yes  No | 1  2 | C5 |
| C4 | Which method are you currently using? | Copper-T/IUD  Injectable  Pill  Condom  Rhythm method  Withdraw  Abstinence  Female sterilization  Male sterilization  ECP  LAM | 01  02  03  04  05  06  07  08  09  10  11 | Skip to Section E |
| D23 | On a scale of 1 to 10, where 10 is extremely important and 1 is not important at all, how important is it you to avoid a pregnancy now? | \|  \|  \| \| --- \| --- \| |  |  |
| E11b | On a scale of 1 to 10, where 10 is extremely important and 1 is not important at all, how important is it for you to avoid a pregnancy now? | \|  \|  \| \| --- \| --- \| |  |  |

**12-month questionnaire**

| C7d | Which method are you currently using? | Copper-T/IUD  Injectable  Pill  Condom  Rhythm method  Withdraw  Abstinence  Female sterilization  Male sterilization  LAM  ECP | 01  02  03  04  05  06  07  08  09  10  11 |  |
| --- | --- | --- | --- | --- |
